# Phytohormone Mediation of Interactions Between Plants and Non-Symbiotic Growth Promoting Bacteria Under Edaphic Stresses

**DOI:** 10.3389/fpls.2019.01368

**Published:** 2019-10-29

**Authors:** Guzel Kudoyarova, Tatiana Arkhipova, Tatiana Korshunova, Margarita Bakaeva, Oleg Loginov, Ian C. Dodd

**Affiliations:** ^1^Ufa Institute of Biology, Ufa Federal Research Centre (RAS), Ufa, Russia; ^2^The Lancaster Environment Centre, Lancaster University, Lancaster, United Kingdom

**Keywords:** plant growth promoting rhizobacteria, plant hormones, drought, salinity, petroleum pollution

## Abstract

The capacity of rhizoshere bacteria to influence plant hormonal status, by bacterial production or metabolism of hormones, is considered an important mechanism by which they promote plant growth, and productivity. Nevertheless, inoculating these bacteria into the plant rhizosphere may produce beneficial or detrimental results depending on bacterial effects on hormone composition and quantity *in planta*, and the environmental conditions under which the plants are growing. This review considers some effects of bacterial hormone production or metabolism on root growth and development and shoot physiological processes. We analyze how these changes in root and shoot growth and function help plants adapt to their growth conditions, especially as these change from optimal to stressful. Consistent effects are addressed, along with plant responses to specific environmental stresses: drought, salinity, and soil contamination (with petroleum in particular).

## Introduction

The capacity of some rhizosphere bacteria (so-called plant growth promoting rhizobacteria—PGPR) to promote plant growth under stressful environments (drought, salinity, suboptimal temperature, toxic metals, pollution with organic substances of man-made origin) is attracting increasing attention (Belimov et al., 2009a; Gerhardt et al., 2009; Paul and Lade, 2014; Vejan et al., 2016; Backer et al., 2018). Microorganisms can directly influence plant growth by synthesizing growth-stimulating hormones (Spaepen and Vanderleyden, 2011; Kudoyarova et al., 2015a; Shi et al., 2017) and metabolizing growth-inhibitory hormones (Belimov et al., 2009a; Glick, 2014). The importance of hormones in mediating such plant/microbe interactions has been highlighted by experiments showing that inoculation with PGPR causes pivotal changes in plant expression of hormone-mediated genes (Lara-Chavez et al., 2015; Ambreetha et al., 2018; Jatan et al., 2018). Changes in plant hormonal status may result from either microbial consumption or production of hormones, or changes in plant hormone metabolism *in planta* (Dodd et al., 2010) that may be induced by volatile substances synthesized by microorganisms (Zhang et al., 2007). In turn, plant hormones such as auxins (Spaepen and Vanderleyden, 2011) affect microbial gene expression following their addition to culture media.

Hormones produced by PGPR are mostly related directly to plant growth promotion, while other effects of PGPR (their capacity to improve mineral nutrition and plant resistance to pathogens and abiotic stress) are considered independently from microbial effects on plant hormonal system in other reviews. Meanwhile, the effects of PGPR on plant hormonal regulation are important not only in directly promoting plant growth, but also in other aspects of PGPR action on plants, such as improving mineral nutrition or plant resistance to biotic stresses. Plant hormones regulate vitally important processes in plants such as mineral nutrition, water relations, resistance to pathogens, and antioxidant functions. Thus, hormone-mediated stimulation of root growth can improve mineral nutrition and water relations (Kudoyarova et al., 2015b and references therein). Furthermore, hormone-mediated stimulation of plant antioxidant systems, such as catalase, ascorbate peroxidase (Zavaleta-Mancera et al., 2007), and CuZn-superoxide dismutase (Tyburski et al., 2009) enzymes, helps protect plants against oxidative stress, which accompanies most detrimental environmental factors (Xia et al., 2015). PGPR can also improve plant mineral nutrition by fixing atmospheric nitrogen or solubilizing phosphates, thereby indirectly affecting phytohormone concentrations *in planta*, since plant hormonal status partially depends on the availability of mineral nutrients (Kudoyarova et al., 2015b and references therein). Thus the capacity of rhizosphere bacteria to influence plant hormonal status is likely involved in most known mechanisms of growth promoting action by PGPR.

However, PGPR effects on plant hormone systems may be either beneficial or detrimental for plants depending on their growth environment, with much literature indicating that inhibition (and not promotion) of plant growth protects plants against stress factors (Chapin, 1991; Chaves et al., 2003; Achard et al., 2006; Bechtold and Field, 2018). This review aims to assess whether the outcome of microbial effects on plant hormonal system changes according to whether plants are grown under either optimal or stressful environments. Special attention is paid to plant/microbe interactions when the soil dries, is salinized, or is contaminated with petroleum. This information may help choose optimal PGPR traits for their use in certain environments. PGPR effects on plant hormonal status is discussed with attention focused on auxins, cytokinins, abscisic acid (ABA) and ethylene. Although PGPRs can produce other plant hormones (e.g. gibberellins or jasmonates), those are not included in this review and interested readers may find information about them in corresponding reviews (Bottini et al., 2004; Van der Ent et al., 2009). Nevertheless, mechanisms of auxins, cytokinins, ABA, and ethylene action may inform general conclusions that are relevant to other plant hormones produced by PGPR.

### Effects of Bacterial Auxins on Root Growth and Development

Changes in root growth and development are most important for adapting plants to either optimal or stressful environments. Microbial acceleration of root growth is very important, since some cultivars are characterized by weak root system development (Ehdaie et al., 2003; Waines and Ehdaie, 2007). New varieties with deeper root systems accessed more of the stored soil moisture at depth than current varieties, thus producing higher yields (Watt et al., 2013). Despite recent efforts, root growth and development still has not been fully exploited as a yield enhancement strategy (Den Herder et al., 2010). Germplasm from many breeding programs has traditionally been evaluated at high levels of mineral nutrition, where enhanced root development is not needed. However, since well-developed roots are needed under conditions of water deficit, many modern plant breeding efforts are focused on vigorous root systems (Comas et al., 2013). Nevertheless, selection of cultivars with specific root traits is a long process, while the use of bacterial preparations potentially enables fast results.

Rhizobacterial stimulation of root growth is mostly considered to be *via* their capacity to synthesize indole acetic acid (IAA—the most common, naturally occurring, plant hormone of the auxin class) (Spaepen and Vanderleyden, 2011), since stimulation of rhizogenesis is one of the best known effects of auxins. Many rhizosphere bacteria can synthesize auxins (Spaepen and Vanderleyden, 2011), with addition of tryptophan to bacterial culture mediа providing a simple method to determine bacterial auxin production (Blinkov et al., 2014). Inoculating canola (*Brassica napus*) plants with mutant strains of *Pseudomomas putida* with decreased synthesis of auxins diminished the growth promoting effect of inoculation on root growth (Patten and Glick, 2002). Similarly, inoculating wheat (*Triticum aestivum*) seedlings with IAA-deficient mutants of the salt tolerant *Pseudomomas moraviensis* decreased root surface area by 13%–38% compared to inoculating with the wild-type strain (Hassan and Bano, 2019). Furthermore, the importance of bacterial auxins for stimulating plant root proliferation was also confirmed in experiments showing that *Azospirillum* mutants deficient in auxin production did not enhance wheat root development (Dobbelaere et al., 1999). Thus mutational analyses of the significance of auxin in plant/microbe interactions gave consistent results across two bacterial genera and different plant species.

Experiments with exogenously applied synthetic auxins showed that their action on root growth may depend on the site of hormone application. Thus addition of auxins to the root tips enhanced lateral root initiation (Casimiro et al., 2001), while shoot application of auxin stimulated lateral root emergence (Reed et al., 1998). In accordance, different plant response may be expected depending on the site of PGPR application that may be achieved either through leaf spraying or seed inoculation (Gunes et al., 2015). Since our article mainly focuses on rhizobacteria, root zone inoculation is mainly considered.

Inoculating wheat plants with the auxin-producing *Paenibacillus illinoisensis* IB 1087 and *Pseudomonas extremaustralis* IB-К13-1А increased root mass and root auxin concentrations (Kudoyarova et al., 2017). Field experiments showed that pre-sowing bacterization of the wheat seeds with auxin-producing (*P. extremaustralis* IB-К13-1А) or phosphate solubilizing strains (*Advenella kashmirensis* IB-К1 and *P. extremaustralis* IB-К13-1А) increased crop yield by 10% to 36%, although the relative significance of each bacterial trait was not clear (Arkhipova et al., 2019). Inoculating seeds with all these strains increased the number of spike bearing tillers compared to uninoculated controls. The number of spikelets in the main spike was greater than in the controls only following *P. extremaustralis* IB-К13-1А inoculation. Treatment with all the strains (except *A. kashmirensis* IB-К1) increased the number of grains and their weight in the main spike. This effect was more pronounced in axillary spikes, where *P. extremaustralis* IB-К13-1А inoculation approximately doubled grain weight and number, with lesser effects following *A. kashmirensis* IB-К1 inoculation. These positive effects of bacterization on wheat productivity occurred following insufficient summer rainfall at the time of grain filling, in agreement with reports of bacterial preparations having greater effects on growth and yield of droughted plants (Kuzmina and Melentev, 2003; Rubin et al., 2017). The cost of seed bacterization per hectare [2400 roubles (about $37) with a sowing density of 3–4 million seeds ha^−1^] was overshadowed by the economic gain of enhanced wheat yield due to PGPR action [22,000 roubles (more than $300) per hectare], suggesting such treatments may be commercially appealing to farmers.

Interestingly, strains capable of either solubilizing phosphates (*A. kashmirensis* IB-К1) or synthesizing auxins (*B. subtilis* IB-21) were less effective than the strain that combined these traits (*P. extremaustralis* IB-К13-1А). Introducing *P. extremaustralis* IB-К13-1А increased soil soluble phosphate concentrations (0.5 N acetic acid extraction) by 10% during the vegetative period compared to uninoculated controls and when bacteria unable to solubilize phosphates were applied (Arkhipova et al, 2019). Inoculating soil with *P. extremaustralis* IB-К13-1А increased root phosphorus concentration by 30%, while increased shoot phosphorus content was due to enhanced shoot biomass and not phosphorus concentration (Kudoyarova et al., 2017). Increased phosphorus uptake by plants inoculated with auxin producing bacteria was due not only to more plant-available phosphorus in the soil, but also to better root development correlating with increased root auxin levels (Kudoyarova et al., 2017). Application of such bacterial preparations may also allow farmers to apply less fertilizers (Adesemoye et al., 2009), thereby decreasing the costs of production.

Increased root mass following PGPR inoculation is also important for phytoremediation of soils contaminated with inorganic and organic pollutants such as toxic metals (Safronova et al., 2006), salinity (Cheng et al., 2007; Habib et al., 2016), and petroleum hydrocarbons (Greenberg et al., 2006). Plants are relatively tolerant of various environmental contaminants and are often used within phytoremediation strategies, but their biomass accumulation (and thus contaminant removal) may be limited in the presence of high contaminant levels (Glick, 2006). PGPRs increase plant tolerance to petroleum pollutants and other stresses (Gerhardt et al., 2009). Alongside the degradation of organic pollutants (Chetverikov et al., 2017, Korshunova et al., 2017), they vigorously promote plant biomass accumulation (Glick, 2003), allowing faster remediation than without PGPR application (Glick et al., 1998). Inoculating oat (*Avena sativa*) plants with *Acinetobacter* sp. increased degradation of total petroleum hydrocarbons in contaminated soil from about 33% to 45% (Xun et al., 2015), while hydrocarbon degradation was more than doubled by inoculating alfalfa (*Medicago sativa*) with *Bacillus* sp. PVMX4 (Benson et al., 2017). These effects are attributed to microbial production and provision of auxins to plants (Huang et al., 2005; Glick, 2006; Benson et al., 2017). Thus microbial auxin production may also offer environmental benefits in remediation programs.

While enhancing root mass may be an appropriate strategy to increase resource acquisition in stressful environments, optimizing root architecture (with the same carbon allocation to the roots) may be a more effective strategy. Here, the complexity of applying auxin-producing bacteria becomes apparent. Auxins stimulate root branching (Laskowski, 2013) to allow capture of light rainfall that fails to infiltrate the soil to a great depth (Hodge, 2010). Although fast root elongation into deeper, moister soil layers is important during soil drying (Xiong et al., 2006), high auxin concentrations may inhibit this process. Rhizobacterial IAA accumulation was significantly correlated with decreased elongation of sugar beet (*Beta vulgaris*) roots following PGPR application (Loper and Schroth, 1986). Applying auxin producing pseudomonad bacteria decreased *Arabidopsis thaliana* root elongation (Zamioudis et al., 2013). Indeed, treating canola seedlings with the IAA-deficient mutant of *Pseudomonas putida* GR12-2 accelerated root elongation, thereby confirming that high auxin concentrations decrease root length (Patten and Glick, 2002). Such root growth inhibition may be detrimental when plants grow in drying soil. Since auxin concentrations decrease in stressed plants due to increased activity of the *GH3* gene involved in conjugating active free auxins (Seo et al., 2009) and activation of IAA-oxidase (Li et al., 2014), drought may sensitize plants to microbial-produced auxins. Thus inoculating common bean (*Phaseolus vulgaris*) with intermediate concentrations (10^7^ CFU ml^−1^) of the auxin-producing *Azospirillum brasilense* strain Cd increased root length of plants grown in drying soil, but not in well-watered plants (German et al., 2000). The effect disappeared at higher inoculum concentrations (10^8^ CFU ml^−1^), suggesting that high concentrations of microbial auxins inhibited root elongation. Thus the optimal level of microbial auxin production to enhance plant growth in drying soil may depend on the relative importance of root branching *versus* elongation in mediating water uptake.

Interestingly, monocotyledonous plants are less sensitive to auxins than dicotyledonous plants (Fedtke, 1982). Morgan and Hall (1962) proposed that the differential sensitivity of monocots and dicots to the synthetic auxin (and herbicide) 2,4-D was caused by differences in the rates of ethylene production following 2,4-D application. Furthermore, root growth of monocotyledonous plants (barley, wheat, and oats) was generally less sensitive than dicotyledonous plants [canola, lettuce (*Lactuca sativa*), tomato (*Solanum lycopersicum*)] to seed treatment with the 1-aminocyclopropane-1-carboxylic (ACC) deaminase containing PGPR *P. putida* GR12-2 or application of the chemical ethylene generator (2-chloroethyl) phosphonic acid (ethephon) (Hall et al., 1996). Differential sensitivity to bacterial auxins has been most clearly shown *via* inhibition of primary root elongation of the dicotyledonous plant *A. thaliana* (Zamioudis et al., 2013), whereas auxin producing bacteria of the *Bacillus*, *Enterobacter*, *Moraxella*, and *Pseudomonas* genera stimulated wheat (monocotyledonous plant) root elongation (Raheem et al., 2017). Nevertheless, auxin producing bacteria may also inhibit wheat root growth (Ali et al., 2009). This diversity of response may result from differences in endogenous auxin content in the studied wheat cultivars. Still, although inhibition of root elongation by auxin producing bacteria is less likely in monocotyledonous than in dicotyledonous plants, root growth inhibition is possible in both cases, which makes plants less drought-tolerant.

**Figure 1:**
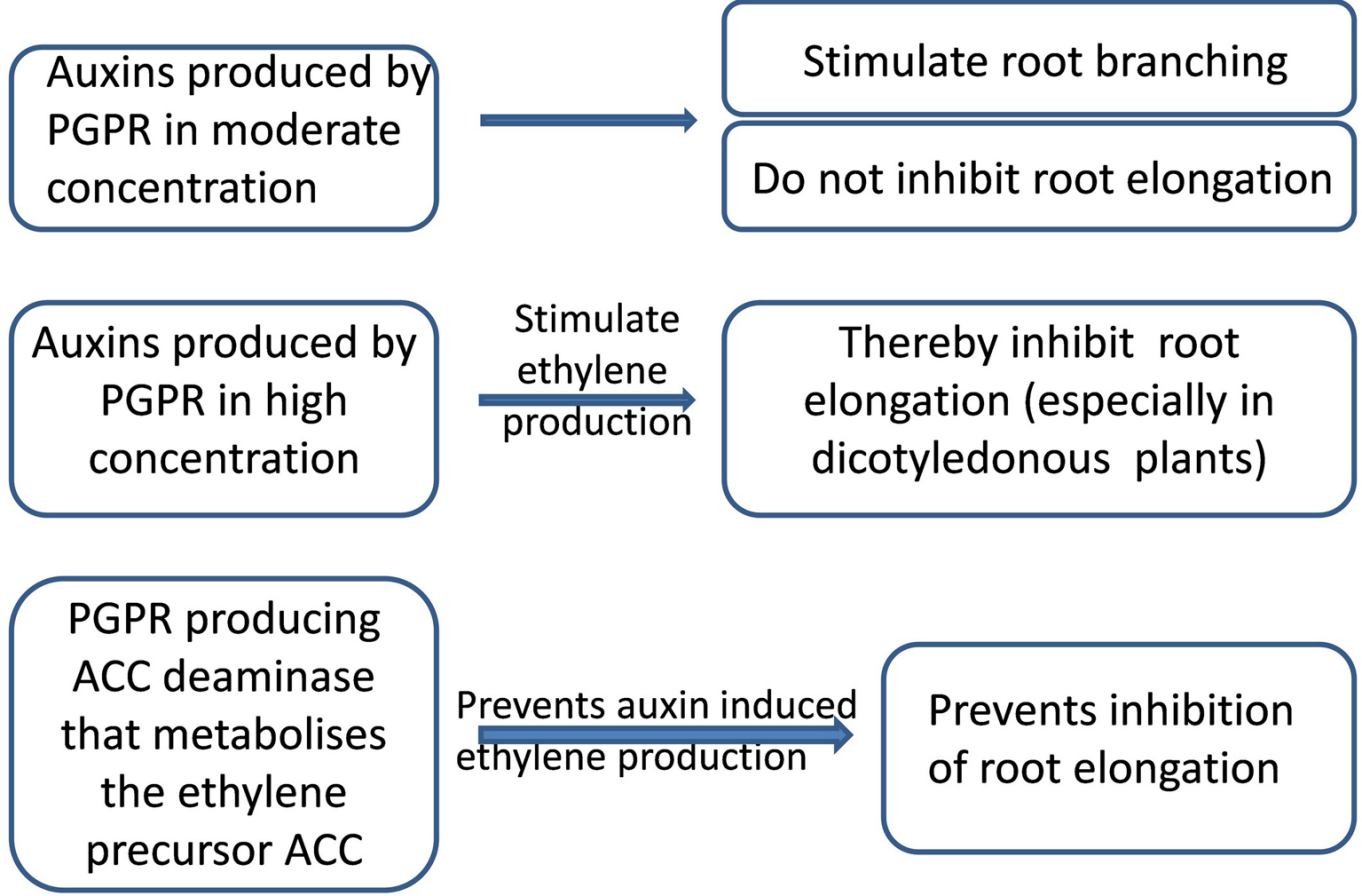
Scheme of bacterial action depending on the level of auxin production and presence of 1-aminocyclopropane-1-carboxylic (ACC) deaminase activity.

Inhibition of root elongation by auxins may seem surprising, since these hormones stimulate shoot cell extension. Auxin effects on root elongation may be explained by their dose-dependent capacity (increasing with auxin concentration—Arteca and Arteca, 2008) to stimulate production of the growth inhibitor ethylene (Glick, 2014). Increased ethylene production may be prevented by bacterial ACC deaminase activity, since ACC is a direct precursor of ethylene (Chen et al., 2013). Bacteria with this enzyme decreased soil and root ACC concentrations (Belimov et al., 2015), thereby lowering ethylene production throughout the plant (Chen et al., 2013) and decreasing its concentration *in planta*. Inoculating pea (*Pisum sativum*) plants with a strain of *Variovorax paradoxus* with high activity of this enzyme attenuated a soil-drying induced increase in xylem ACC concentration (Belimov et al., 2009b). The ACC deaminase mediated decrease in ethylene synthesis enhances root elongation, despite potentially inhibitory high concentrations of auxins (**Figure 1**). *Enterobacter cloacae* UW4 capable of synthesizing both IAA and ACC deaminase promoted root elongation of canola plants, while its ACC deaminase minus mutant failed to influence root growth suggesting this enzyme is required for bacteria to exert beneficial effects on root elongation (Li et al., 2000). Patten and Glick (2002) suggested that IAA and ACC deaminase work in concert to stimulate root elongation. Thus the capacity of rhizobacteria to produce both auxins and ACC deaminase becomes especially important under unfavorable conditions (Glick, 2014).

Bacteria producing ACC deaminase prevent not only auxin-induced, but also stress-induced, synthesis of ethylene, by consuming ACC. In practice, ACC deaminase-containing plant growth promoting bacteria have been used to protect plants against growth inhibition caused by the presence of organic toxicants and total petroleum hydrocarbons (Huang et al., 2005; Hong et al., 2011), a variety of different metals (Tiwari and Lata, 2018), high salt (Cheng et al., 2007; Singh and Jha, 2016), and drought (Mayak et al., 2004; Niu et al., 2017).

Thus applying auxin-producing PGPR may produce different outcomes under stressful and optimal conditions. Although microbial auxins can stimulate root branching, which should enhance water and nutrient capture, high concentrations of microbial auxins can inhibit root elongation which may be detrimental under drought, when long roots are necessary for extracting water from deep soil layers. Nevertheless, this potentially negative side effect of microbial auxins may be prevented by bacteria also having ACC deaminase activity.

### Plant Response to Cytokinins Produced by Bacteria

The capacity of PGPR to synthesize cytokinins has been studied much less frequently than auxin production, although almost every review on PGPR mentions microbial production of cytokinins. While cytokinins undoubtedly have direct impacts on various plant processes (e.g. stimulating cell division), often the balance between auxin and cytokinin levels is considered a key regulator of plant organogenesis and root architecture. Since some PGPR are able to produce both of these hormones (Vacheron et al., 2013), tissue auxin to cytokinin ratio can be important in determining plant response to rhizobacterial inoculation.

Almost all known cytokinins were identified in the growth media of *Paenibacillus polymyxa* after their separation by immunoaffinity chromatography and final identification by gas chromatography–mass spectrometry (Timmusk et al., 1999). Microbial cytokinin production was suggested to stimulate plant growth, although not directly studied (Timmusk et al., 1999). A quarter of the pseudomonads isolated from rhizospheres of different crops (*Pennisetum glaucum*, *Helianthus annuus*, *Zea mays*) grown under 25 arid and semi-arid locations across India were capable of producing cytokinins, when grown under osmotic stress (25% PEG 6000) (Sandhya et al., 2010). Of 70 rhizobacterial strains isolated from the *Coleus* rhizosphere, three (*Pseudomonas stutzeri*, *Stenotrophomonas maltophilia*, and *P. putida*) produced cytokinins (Patel and Saraf, 2017). Since these bacteria also synthesized other hormones (auxins and gibberellins), it was difficult to determine the mechanistic basis of plant growth promotion. More convincing evidence of cytokinin involvement in the plant growth promoting effect of *Bacillus megaterium* was obtained using the triple cytokinin receptor *CRE1-12/AHK2-2/AHK3-3* knockout mutant of Arabidopsis, in which plant development was insensitive to inoculation (Ortíz-Castro et al., 2008). Microbial cytokinin production was identified as a key determinant of the ability of *Pseudomonas fluorescens* G20-18 to regulate *Arabidopsis* development, since inoculation with G20-18 cytokinin deficient loss-of-function mutants had no effect on the plant development (Großkinsky et al., 2016). Thus microbial cytokinin production and plant sensitivity to cytokinins are both necessary for microbial stimulation of plant growth in certain plant/microbe interactions.

**Figure 2:**
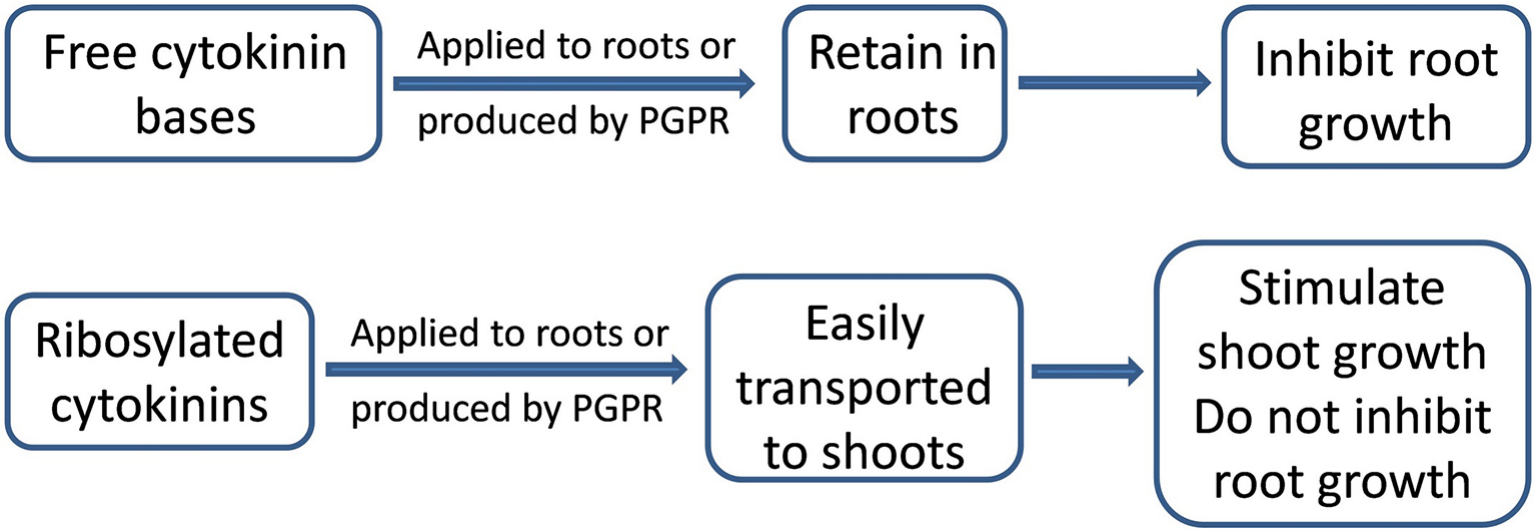
Effects cytokinins on root and shoot growth. Free cytokinin bases retain in the roots and inhibit their growth, while ribosylated cytokinins flow to the shoots and stimulate their growth without inhibiting root growth.

Introducing *B. subtilis* IB 22 (with high cytokinin production) into wheat and lettuce rhizospheres increased leaf area (Arkhipova et al., 2005), since cytokinins stimulate shoot cell division and elongation (Werner et al., 2003). At the same time, cytokinins can inhibit root growth (Werner et al., 2010). *Bacillus amyloliquefaciens* UCMB5113 inhibited primary root growth of *Arabidopsis*, which may be due to bacterial cytokinin production and increased root cytokinin levels, or the increased auxin levels that were also detected in colonized roots (Asari et al., 2017). Nevertheless, introducing a bacterial suspension of *B. subtilis* IB 22 into wheat rhizospheres did not decrease root biomass accumulation (Kudoyarova et al., 2014a). Following rhizobacterial inoculation, cytokinins (predominantly zeatin riboside) appeared in the roots, but later shoot cytokinin accumulation occurred while root cytokinin concentration declined (Arkhipova et al., 2005). Since *B. subtilis* IB 22 produced ribosylated cytokinin forms that are readily transported out of the roots, root cytokinin accumulation did not occur and the roots grew normally. Thus introducing cytokinin-producing microbes into the rhizosphere may not necessarily inhibit root growth if they are transported to the shoots (**Figure 2**).

Comparing the root/shoot cytokinin distribution of wheat plants, to which either free cytokinin bases or their ribosides were root-applied, explained how cytokinins were not retained in the roots of inoculated plants (Korobova et al., 2013). Moreover, inhibiting root cell cytokinin uptake by the protonophore, carbonyl cyanide m-chlorophenylhydrazone; increased xylem sap cytokinin concentrations and their flow to the shoot (Kudoyarova et al., 2014b). Thus free bases of cytokinins were actively absorbed by root cells (Korobova et al., 2013). Unlike free zeatin, more cytokinins accumulated in the shoots than in the roots of plants treated with zeatin riboside applied to their roots. The results, showing rapid export of root-derived ribosides of cytokinins to the shoot (Korobova et al., 2013), explain how riboside-producing bacteria do not inhibit root growth, but instead stimulate leaf growth (Arkhipova et al., 2005).

Although cytokinin-producing bacteria may limit root proliferation thereby minimizing salt uptake, the role of bacterial cytokinins in salt stress tolerance is largely unknown as there have been few studies (Ilangumaran and Smith, 2017). Nevertheless, whether rhizobacterial cytokinin production influences plant drought response was evaluated, since increased leaf area and greater stomatal opening mediated by cytokinins could accelerate soil moisture depletion (Davies et al., 2005). Inoculating well-watered plants with different cytokinin producing *B. subtilis* strains increased shoot ABA concentrations of lettuce (Arkhipova et al., 2007) and *Platycladus orientalis* (Liu et al., 2013) by 2.1-fold and 1.8-fold respectively. Shoot total cytokinin concentrations were increased by 1.8-fold and 2-fold respectively. With similar fold-changes in these two phytohormones having antagonistic effects on stomatal opening (Dodd, 2003), stomatal conductance of well-watered, inoculated plants was similar or slightly (15%) increased compared to uninoculated plants. Since inoculation increased shoot biomass, these plants were exposed to more rapid soil drying (due to greater transpiration by larger leaves) after withholding water, then once a threshold soil water content was achieved, it was maintained *via* daily, suboptimal irrigation. Inoculation alleviated the impacts of soil drying on shoot total cytokinin concentrations in both studies, but eliminated (Arkhipova et al., 2007) or magnified (Liu et al., 2013) the effects of soil water deficit on shoot ABA accumulation. Despite these contrasting effects on shoot ABA accumulation, inoculation generally had no effect on soil-drying induced stomatal closure. Nevertheless, both studies demonstrated that inoculation with cytokinin-producing bacteria enhanced plant growth in drying soil, even if the relative effects of changes in shoot ABA and cytokinin concentrations were not explored.

Both salinity (Albacete et al., 2008) and drought (Kudoyarova et al., 2007) decreased foliar cytokinin concentrations, which may inhibit leaf growth. Introducing cytokinin producing bacteria of *B. subtilis* IB 22 strain into the rhizosphere of lettuce plants increased cytokinin contents (and leaf area) of both well-watered and droughted plants (Arkhipova et al., 2007). Although leaf growth inhibition can protect plants against terminal drought stress (Avramova et al., 2015) by conserving water for use during reproductive development, it almost inevitably decreases crop yield under more moderate conditions. Moreover, leaf growth inhibition may delay canopy closure which limits direct evaporation from moistened soil (Tardieu, 2005). Consequently, accelerated leaf growth by microbial cytokinins may be advantageous in dryland agriculture if the soil is rapidly covered following planting to minimize evaporation from the soil. Under conditions of moderate drought, a pre-sowing treatment of wheat seeds with the cytokinin-producing *B. subtilis* IB 22 promoted early canopy closure and increased yield by 40% (Wilkinson et al., 2012). This effect of *B. subtilis* IB 22 on wheat yield was repeated in several (dry) years and comparable to the effects of auxin producing bacteria (Arkhipova et al., 2019). Thus microbial cytokinin production may provide agronomically useful seed treatments under conditions of moderate drought.

Alfalfa tolerance to severe drought stress (watering ceased for 2 weeks until non-inoculated plants died) was increased by inoculating plants with engineered *Sinorhizobium* strains overproducing cytokinins due to expression of the *Agrobacterium ipt* gene under the control of different promoters (Xu et al., 2012). Most of the alfalfa plants inoculated with engineered strains survived under conditions of severe stress, which was attributed to increased expression of antioxidant enzymes and decreased level of reactive oxygen in inoculated stressed plants. These findings suggest that engineered *Sinorhizobium* strains synthesizing more cytokinin could improve the tolerance of alfalfa to severe drought stress.

Thus cytokinins produced by PGPR are likely to promote plant growth, thereby enhancing productivity either under normal or stress conditions. The potential of cytokinins to augment stomatal opening is counteracted by plant ABA accumulation to prevent excessive water losses, while cytokinins-induced increase in leaf size accelerates canopy closure to prevent evaporation of water from the soil. Nevertheless, under severe drought the capacity of cytokinins to inhibit root elongation (as with auxins) may limit water extraction from deep soil layers. Still, the inhibitory action of bacterial cytokinins on the root growth may be prevented, when they are produced in ribosylated forms that are readily exported to the shoots and not retained in the roots.

### The Role of ABA in Plant/Bacteria Interactions

Changes in plant ABA status may also be important in mediating plant/microbe interactions, by antagonizing effects of microbial cytokinin production on stomatal conductance as discussed above (Arkhipova et al., 2007; Liu et al., 2013) and also having direct effects. Moreover, plant ABA status can mediate the outcome of interactions with PGPR, as when *B. megaterium* inoculation stimulated growth of wild type tomato (*S. lycopersicum*), but inhibited shoot biomass of the ABA-deficient tomato mutants *flacca* and *sitiens* (Porcel et al., 2014). In the absence of microbial effects on stomatal conductance (and presumably photosynthesis) in all genotypes, changes in growth were attributed to microbial stimulation of shoot ethylene production. Microbial inoculation increased ethylene production of WT and *flacca* plants by 2.2-fold and 4.1-fold respectively, with the excessive ethylene accumulation triggered by the PGPR in *flacca* associated with growth inhibition rather than growth promotion. Interestingly, uninoculated *flacca* plants produce at least twice as much ethylene as WT plants (Sharp et al., 2000; Dodd et al., 2009), further suggesting the importance of ABA/ethylene interactions in growth regulation. In contrast, the ABA-metabolizing *Rhodococcus* sp. P1Y and *Novosphingobium* sp. P6W had similar effects on root and shoot biomass of ABA-deficient mutant *flacca* and WT plants grown *in vitro*, probably since ethylene was not involved in regulating these plant/microbe interactions (Belimov et al., 2014). Based on these studies with ABA-deficient mutants, it is difficult to be certain whether ABA status is the primary determinant of these plant/microbe interactions.

Moreover, microorganisms can synthesize ABA (Cohen et al., 2009; Cohen et al., 2015; Shahzad et al., 2017) that should alter ABA-mediated processes due to plant uptake of microbially produced hormone. Otherwise microbes can influence expression of plant genes (e.g. NCED3, ABA2 and ABA3) responsible for ABA biosynthesis *in planta* (Vargas et al., 2014). Thus inoculation of sugarcane (*Saccharum officinarum*) roots with *Gluconacetobacter diazotrophicus* had different transcriptome profiles (for genes responsible for ABA biosynthesis and signaling) from uninoculated plants, with *G. diazotrophicus* activating ABA-dependent signaling genes in the shoots, which may confer drought resistance (Vargas et al., 2014). Nevertheless, it can be difficult to ascribe changes in plant ABA status to direct microbial ABA biosynthesis or to changes in ABA synthesis *in planta* due to changes in plant water status resulting from plant growth promotion. In some reports, ABA concentration in bacterial culture media was related to actual changes in ABA content *in planta* (Yasmin et al., 2017). *Bacillus pumilus* produced five times more ABA than *Pseudomonas* sp. *in vitro*, thereby greatly increasing relative water content and osmotic potential of inoculated plants. Microbial ABA production by *Azospirillum lipoferum* increased ABA concentrations *in planta* (Cohen et al., 2009; Cohen et al., 2015), preventing a drought-induced decline in relative water content of inoculated maize (*Z. mays*) seedlings. Although ABA accumulation was related to some physiological effects associated with PGPR inoculation (see below), a causal relation between them was not frequently supported experimentally.

ABA protects plants from dehydration by stimulating expression of dehydrins (Shakirova et al., 2016). In accordance with this effect of ABA, genes involved in the synthesis of dehydrins [late embryogenesis abundant (LEA) proteins] were upregulated in maize plants inoculated with *P. putida* strain FBKV2 (SkZ et al., 2018). FBKV2 inoculation enhanced SnRK2 family proteins, which facilitated transcription of ABA-responsive genes, thereby conferring drought tolerance. *Arabidopsis* plants inoculated with ABA producing *A. brasilense* showed less lipid damage quantified by malondialdehyde levels. Although this effect was attributed to ABA-induced antioxidative defense mechanisms (e.g. increased content of phenolic compounds that scavenge free radicals), a simpler explanation may be that ABA-induced stomatal closure improved leaf water relations and thus minimized oxidative stress (Cohen et al., 2015). Thus it can be difficult to separate direct molecular effects of microbial ABA production from indirect effects of enhancing shoot water status.

Furthermore, soil drying can increase ABA concentrations in the soil solution (Hartung et al., 2002) and stimulate root-to-shoot signaling of ABA prior to any changes in shoot water status (Davies et al., 2005). Bacteria may alter this signaling, by synthesizing ABA under stress conditions (e.g. in some strains of *B. pumilus*) (Forchetti et al., 2007) or metabolizing ABA present in the soil solution, thereby decreasing ABA concentrations *in planta* (Belimov et al., 2014). Despite being unable to metabolize ABA *in vitro*, *V. paradoxus* 5C-2 decreased root ABA concentrations and accumulation by 40%–60% in pea, which was related to bacterial ACC deaminase (Jiang et al., 2012). Since an important function of ABA is to restrict ethylene production *in planta* (Sharp et al., 2000), microbial limitation of plant ethylene production seems to diminish the need for high ABA levels.

**Figure 3:**
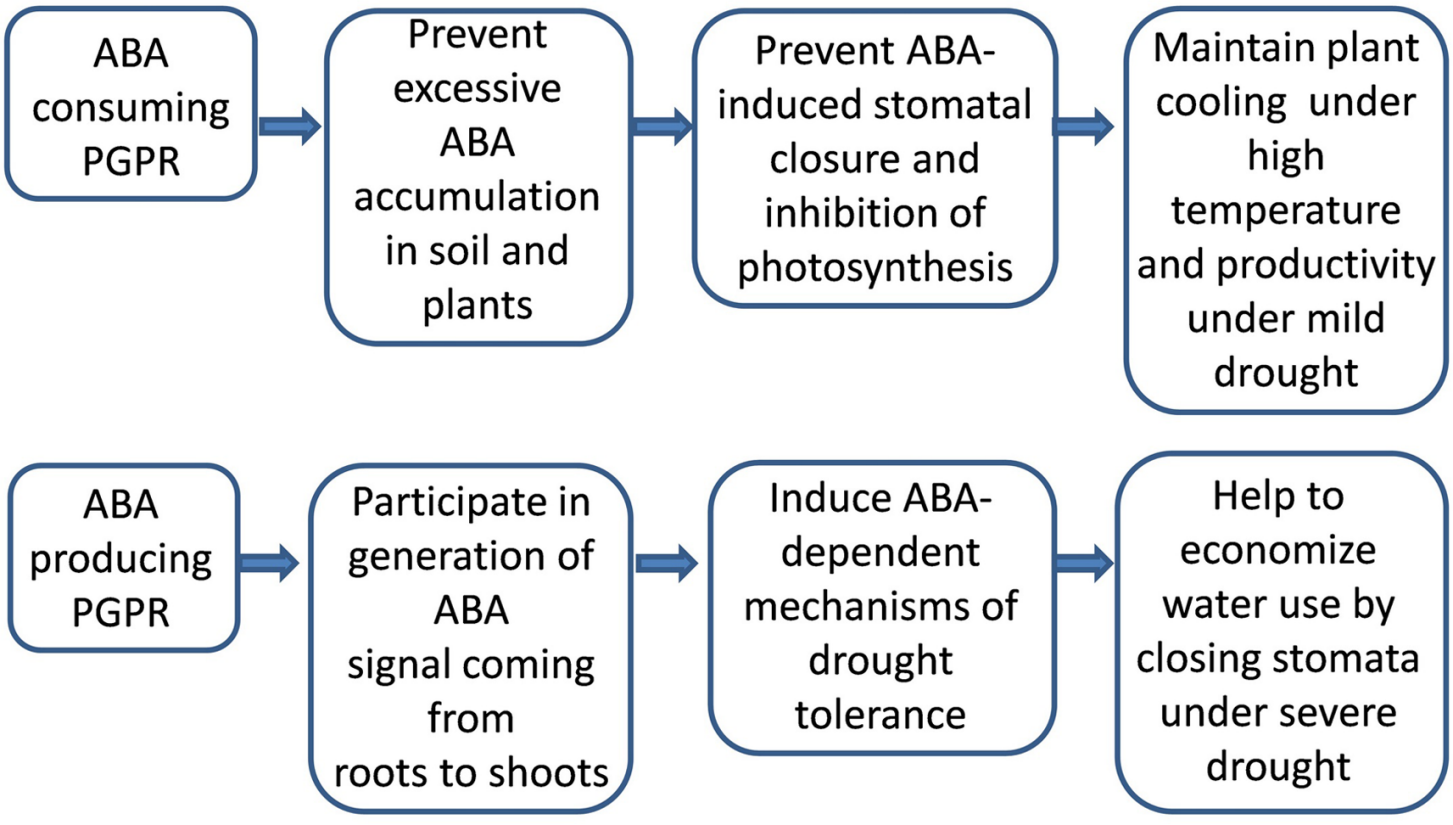
Outcome of action of PGPRs either producing or consuming abscisic acid (ABA) depends on conditions of plant growth.

Since plant growth promotion by rhizobacteria occurred with both increased [e.g. *Bacillus licheniformis* Rt4M10 and *P. fluorescens* Rt6M10 (Salomon et al., 2014)] and decreased ABA concentration [*B. subtilis* GB03 (Zhang et al., 2008)], it is difficult to conclude that ABA was involved in growth regulation. The growth response to the microbial induced changes in ABA content may vary according to tissue water status: with increased ABA concentration maintaining root growth under low water potential, but decreased ABA concentrations maintaining root growth under high water potential (Sharp et al., 1994). Nevertheless, any bacterial-mediated changes in ABA level may affect stomatal conductance. Stomatal closure in inoculated plants maintained leaf water relations under soil water deficit (Salomon et al., 2014). In contrast, stomatal conductance increased under optimal conditions due to decreased ABA content *in planta* (due to emission of volatile compounds by *B. subtilis* GB03), thereby maintaining photosynthesis (Zhang et al., 2008). This contradiction (beneficial effect of both increased and decreased stomatal conductance induced by PGPR) is because stomatal closure both restricts water loss while inhibiting photosynthesis (**Figure 3**). The agronomic outcome of this trade-off between economizing water use and utilizing CO_2_ depends on soil water availability (Ewers, 2013).

ABA not only closes stomata, but also can also increase hydraulic conductance of plant tissues, which can be explained by ABA’s effect on the activity of water channels aquaporins (Kaldenhoff et al., 2008). Although inoculation with *B. megaterium* activated expression of the genes coding for water channels (Marulanda et al., 2010), it is uncertain whether this change was ABA-mediated as ABA levels were not quantified. Indeed, *B. megaterium* decreased root ABA concentrations of the *flacca* mutant (Porcel et al., 2014). Nevertheless, immunohistochemistry of ABA and aquaporin contents in root epidermal cells showed that ABA application to nutrient solution increased the level of HvPIP2;2 and HvPIP2;1 aquaporins in these cells, coincident with increased ABA concentrations (Sharipova et al., 2016). Thus bacterial ABA production (known to increase under osmotic stress—Forchetti et al., 2007) may theoretically mediate root aquaporin activity, although whether the PGPR enhance root ABA concentrations sufficiently to allow this has not been assessed.

## Conclusion

To conclude, the benefits of applying rhizosphere bacteria capable of synthesizing or consuming plant hormones will depend on soil conditions. Bacterial auxin synthesis may stimulate root growth and increase plant productivity under favorable conditions or mild stress. Under more severe stress, this trait should be combined with ACC deaminase activity in the same strain or another bacterial species in the consortium. To prevent inhibitory effects of bacterial cytokinins on root growth, it is important that microbes produce cytokinins in ribosylated forms that are readily exported to the shoot to stimulate cell division and expansion. Bacterial mediation of plant ABA levels merits further study, but decreasing ABA content seems beneficial under moderate stress, while ABA-producing bacteria are beneficial under severe stress. Inter-cultivar variation in intrinsic or stress-induced hormone status (Quarrie, 1981; Sanguineti et al., 1999; Valluru et al., 2016) or hormone sensitivity (Ibort et al., 2017) should be considered when choosing optimal traits for bacterial preparations. There is still limited knowledge about how plants integrate their intracellular signaling in response to multiple phytohormones produced by PGPR, and how these interact with endogenous plant pathways. Such information seems necessary to predict the outcomes of PGPR inoculation, especially when such organisms produce multiple phytohormones. Moreover, the role of hormones in mediating plant/microbe interactions will be important in determining the success (or otherwise) of phytoremediation of salt-affected or oil contaminated soils. However, these interactions require further study to determine whether such biotechnological approaches can deliver predictable outcomes.

## Author Contributions

All authors listed have made a substantial, direct, and intellectual contribution to the work, and approved it for publication. GK – arranged the plan of the review, was responsible for sections concerning hormonal aspects of the review, collected and integrated sections written by other authors, TA was responsible for the material concerning salt stress, TK was responsible for microbial aspects of the review, MB added information about remediation of soils contaminated with iol, OL integrated microbial components of the review, ID was responsible for physiological aspects of water relations and drought resistance and checked English of the text.

## Funding

The work was granted by Russian Foundation for Basic Research N 18-29-05025/18 and N 18-04-00577/18.

## Conflict of Interest

The authors declare that the research was conducted in the absence of any commercial or financial relationships that could be construed as a potential conflict of interest.
